# Soil properties explain tree growth and mortality, but not biomass, across phosphorus-depleted tropical forests

**DOI:** 10.1038/s41598-020-58913-8

**Published:** 2020-02-10

**Authors:** Jennifer L. Soong, Ivan A. Janssens, Oriol Grau, Olga Margalef, Clément Stahl, Leandro Van Langenhove, Ifigenia Urbina, Jerome Chave, Aurelie Dourdain, Bruno Ferry, Vincent Freycon, Bruno Herault, Jordi Sardans, Josep Peñuelas, Erik Verbruggen

**Affiliations:** 1 Climate and Ecosystem Science Division, Lawrence Berkeley National Laboratory, Berkeley, 94720 California USA; 2 PLECO (Plants and Ecosystems), Department of Biology, University of Antwerp, Wilrijk, 2610 Belgium; 3 Center for Ecological Research and Forestry Application, 08193 Cerdanyola del Vallès, Catalonia Spain; 4 Consejo Superior de Investigaciones Científicas, Global Ecology Unit CREAF-CSIC-UAB, 08193 Bellaterra, Catalonia Spain; 5 INRAE, UMR, Ecofog, AgroParisTech, CIRAD, CNRS, Université de Antilles, Université de Guyane, 97310 Kourou, France; 6 Paul Sabatier University, CNRS, Toulouse, France; 7 CIRAD, UMR Ecofog (AgroParisTech, INRAE, CNRS, Univ Antilles, Univ Guyane), Campus Agronomique, 97310 Kourou, French Guiana; 8 Université de Lorraine, AgroParisTech, INRAE, Silva, 54000 Nancy, France; 9 CIRAD, UPR Forêts et Sociétés, F-34398 Montpellier, France; 10 UPR Forêts et Sociétés, Université de Montpellier, Montpellier, France; 11 Institut National Polytechnique Félix Houphouët-Boigny, Yamoussoukro, Ivory Coast

**Keywords:** Climate-change ecology, Biogeochemistry, Carbon cycle

## Abstract

We observed strong positive relationships between soil properties and forest dynamics of growth and mortality across twelve primary lowland tropical forests in a phosphorus-poor region of the Guiana Shield. Average tree growth (diameter at breast height) increased from 0.81 to 2.1 mm yr^−1^ along a soil texture gradient from 0 to 67% clay, and increasing metal-oxide content. Soil organic carbon stocks in the top 30 cm ranged from 30 to 118 tons C ha^−1^, phosphorus content ranged from 7 to 600 mg kg^−1^ soil, and the relative abundance of arbuscular mycorrhizal fungi ranged from 0 to 50%, all positively correlating with soil clay, and iron and aluminum oxide and hydroxide content. In contrast, already low extractable phosphorus (Bray P) content decreased from 4.4 to <0.02 mg kg^−1^ in soil with increasing clay content. A greater prevalence of arbuscular mycorrhizal fungi in more clayey forests that had higher tree growth and mortality, but not biomass, indicates that despite the greater investment in nutrient uptake required, soils with higher clay content may actually serve to sustain high tree growth in tropical forests by avoiding phosphorus losses from the ecosystem. Our study demonstrates how variation in soil properties that retain carbon and nutrients can help to explain variation in tropical forest growth and mortality, but not biomass, by requiring niche specialization and contributing to biogeochemical diversification across this region.

## Introduction

**Figure 1:**
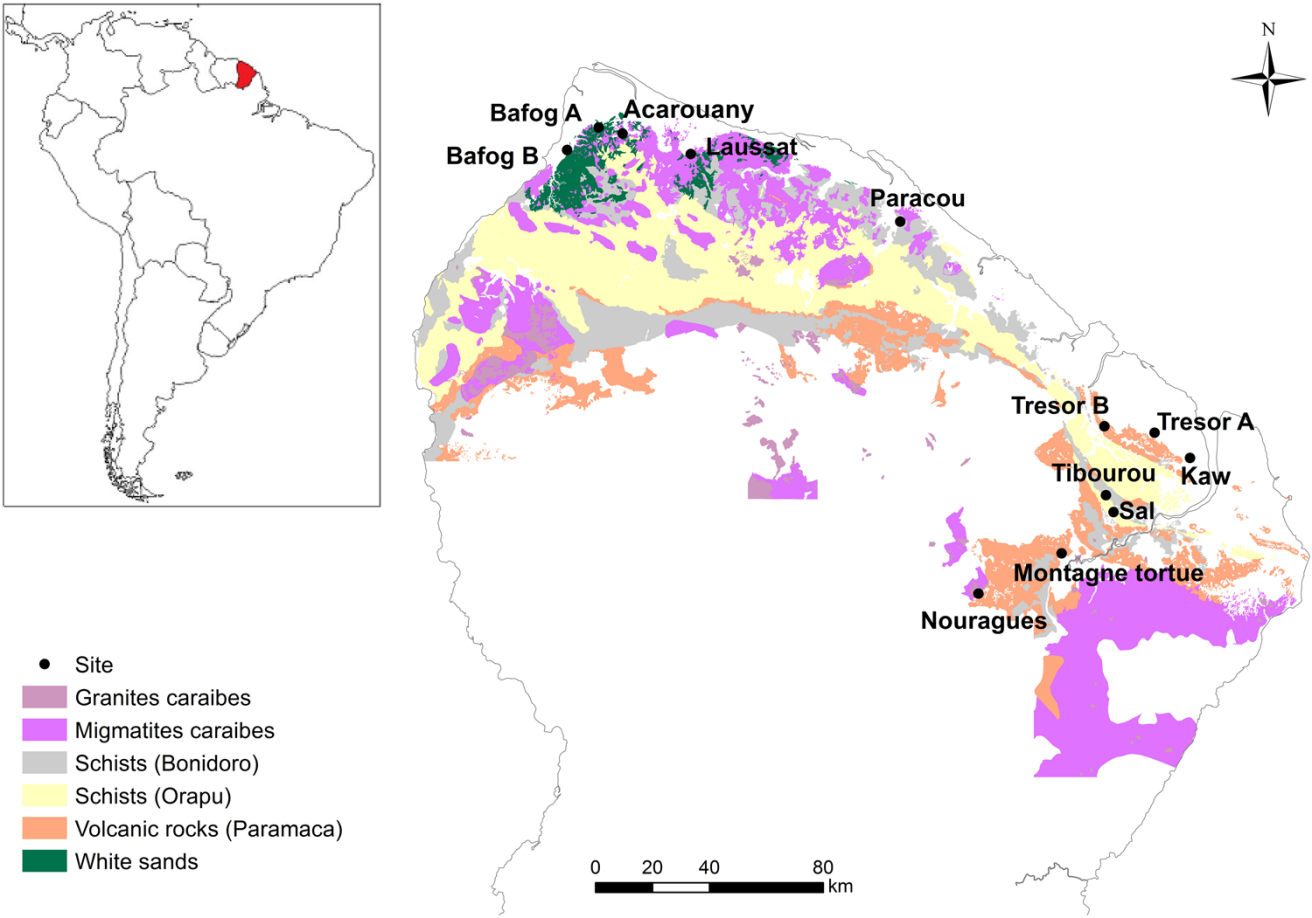
Location of French Guiana in South America (insert) and location of study sites within French Guiana. Geological substrates underlying the region are indicated by color. The Paramaca series is constituted by sedimentary rocks, metamorphic rocks and volcanic rocks of different composition (e.g. andesites, basalts, dacites). White sands are low consolidated sandstones made of quartz. Map created using ArcGIS Version 10.3 https://desktop.arcgis.com/en/^55^.

Amazon tropical forests account for a large fraction of the global forest carbon (C) sink, yet uncertainties about the capacity for that sink to sustain in the future is a major concern for C cycle-climate projections^1,2^. Phosphorus (P) is primarily derived from rocks and becomes depleted over millions of years due to landscape weathering in the tropics^3–5^. One of the most significant illustrations of this is found in the Guiana Shield, an ancient landscape region neighboring the Amazon basin that spans a third of Amazonia and has some of the lowest measured soil P contents among the South and Central American rain forests^3,6^. Guiana Shield forests are characterized by high biodiversity and high aboveground biomass despite their low P soils. Initial mapping of the distribution of geological substrates across the northern region of French Guiana reveals vast belowground geological diversity as well^6^ (Fig. 1). A better understanding of what drives variation in forest dynamics, nutrient availability and C cycling in these forests is a biogeochemical link needed to upscale from geology to earth systems in these critical regions that are being impacted by increasing pressures from deforestation and climate change^7^.

The Guiana Shield is an ancient Precambrian geological formation in northeast South America that has undergone 1.7 billion years of soil formation. The northern region of French Guiana has been forested throughout the Holocene^8^ providing ample time for ecosystem development to what could be considered a steady state. In younger, more nutrient-rich tropical regions, individual tree species trait-based responses have been found to explain the variable forest responses to fertilization^9–11^. Here, we focus instead on how variation in soil properties of texture and mineralogy may drive forest stand-level dynamics, a better understanding of which could aid in the development of coupled belowground-aboveground C-nutrient ecosystem models^12^.

In contrast to the quartz sand coarse soil fraction, the finer soil fraction of the highly weathered soil mantle of the Guiana Shield is dominated by minerals that protect soil organic matter from mineralization by microorganisms through sorption and/or entrapment of organic matter in small microaggregates^13–15^. Among these minerals, Aluminum (Al) and Iron (Fe) oxides and hydroxides, such as Gibbsite (Al hydroxide) and Goethite (Fe hydroxide), have a greater affinity for organic matter than clay minerals, thanks to their large specific surface area^16^. These minerals also constrain the nutrient status in low pH soils through their ability to remove inorganic anions, such as phosphate or nitrate, from solution, forming inner or outer sphere complexes^17,18^. Together with these very stable hydroxides, Kaolinite 1:1 clay, is the most abundant mineral of the fine soil fraction in highly weathered soils. Despite being less reactive than oxides, Kaolinite can present an important anion exchange capacity at low pH^19^, contributing to lower extractable P concentrations traditionally used as an indicator of readily plant accessible P^20–22^. Thus, the same clays and oxides that lead to greater soil organic matter storage also occlude P into less accessible forms^23^, reducing its mobility and making uptake of P resources from the soil more difficult.

However, recent studies suggest that soil P in non-readily extractable forms can be accessed by plants via a myriad of methods such as enzymes^24^, cluster and dauciform roots^25^, carboxylate production^26^, or association with symbiotic mycorrhizal fungi that aid in P uptake^27,28^. We are interested in exploring how interactions between soil substrate properties, organic matter, and microbe-plant symbioses could ultimately be connected to forest carbon cycling properties of tree growth and mortality.

There is clear evidence for natural gradients in soil nutrient availability impacting forest productivity across large-scale gradients spanning chrono-sequences in geologic and pedogenic timescales^11,29,30^. However, support for this paradigm within more limited biogeographic regions of the tropics remains elusive^6,31^. This could be due to the fact that regions spanning latitudes differ not only in geologic history, but also in climate and dominant vegetation structures. A previous examination of Guiana Shield forests found that sites with equally high rainfall can vary massively in growth and turnover rates^6^. The limited number of sites in our study (twelve), along with confounding variation in soil properties, makes it difficult to draw conclusions about how the precipitation gradient across the Guiana Shield may impact forest properties. Instead, we focus on how soil physico-chemical and biological properties that affect soil organic matter and P retention may also impact aboveground dynamics, and could shed light on plant-soil drivers of ecosystem carbon cycling and forest diversity.

Our research was driven by three main hypotheses. First, that soil mineralogy has a strong influence on soil C, nitrogen (N) and P variation between Guiana Shield forests due to the wide range in geology and soil texture in the region. Second, that the prevalence of mycorrhizal fungi increases as soil P declines as a reflection of the role they play in helping plants to obtain P when it is occluded. Third, that aboveground forest biomass, growth, and mortality are related to soil properties that allow for the provisioning of nutrients and organic matter retention in the soil. Thus, we explore here how patterns in soil mineral matrix retention of C and nutrients, along with fungal symbioses that facilitate access to mineral-bound nutrients, relate to patterns of forest biomass and life history strategies across the lowland humid tropical forests of French Guiana.

We surveyed twelve undisturbed, well-drained, lowland tropical forest sites across a 200 km region in French Guiana, on the Guiana Shield, for soil, leaf litter and fungal community characterization, and compared that data to a long-term forest monitoring dataset from ten of the same sites (Fig. 1)^6^. Nine of the ten forest monitoring sites had multiple years of tree growth data from 1-ha, long-term monitoring plots, including data from >34,000 trees to assess tree growth and tree mortality rates. In July 2015, we sampled soils from 0–15, and 15–30 cm depths at five replicate spots within a 20 × 20 plot located in the center of the ten 1-ha forest monitoring plots and at the two additional sites. Here, we synthesize this data from these lowland tropical forests covering a broad range of soil textures and metal-oxide content (Table 1) to examine how soil mineralogy varies with the stock and forms of C, N and P in soils, as well as the relative abundance of mycorrhizal fungi in soils. We also examine how these belowground characteristics in turn co-vary with aboveground forest community characteristics of tree growth and mortality rates, aboveground biomass and nutrient turnover.

**Table 1:** Site descriptions and soil properties from the top 0–15 cm.

Site Name	Precipitation (mm/year)^+^	Grain size			Mineralogy**			Species Richness (ha^−1^)°
		% Sand	% Silt	% Clay	% Quartz	% Gibbsite + Iron Oxide	% Kaolinite	
Acarouany (ACA)	2447	67	17	16	50	3	47	133
Bafog-A (BAA)	2527	79	13	8	68	0	32	116
Bafog-B (BAB)	2527	83	6	11	77	1	22	100
Kaw (KAW)	4012	14	51	35	16	52	32	157
Laussat (LAU)	2521	98	2	0	100	0	0	48
Montagne Tortue (MON)	4358	11	51	38	7	28	51	166
Nouragues-B4 (NOU)	2874	80	14	6	54	7	39	162
Paracou-B4 (PAR)	3141	64	18	18	80	0	20	99
*Sal (SAL)	3996	69	30	1	100	0	0	NA
Tibourou (TIB)	3996	15	49	36	13	39	52	NA
Trésor-A (TRA)	3458	15	43	42	16	52	32	144
*Trésor-B (TRB)	3358	17	57	26	24	1	75	NA

Values are the result of analysis of a composite soil sample from the five replicated spots within each 20 × 20 plot.

*Sites sampled in 2015 but not included in Guyafor forest monitoring study so not included in any analyses using aboveground biomass, tree growth or mortality. ^+^Annual precipitation values are an average of values from 2000–2009 reported from the closest weather station of Météo France.

**Minerology is based on a semi-quantitative XRD analysis, not included in this table are other undetermined phases, likely amorphous oxides.

°Species Richness is the number of tree species per hectare rarefied to 403 identified trees, which allows for comparison in light of variation in the number of unidentified trees between plots.

## Results

A principle components analysis of soil properties, mycorrhizal fungi, and forest dynamics revealed a clear separation of the French Guiana lowland tropical forests sites, with PC1 explaining 46% of the variation in the data (Supplemental Fig. 1). PC1 was largely driven by belowground properties of soil clay content, Fe and Al oxides, and total P, as well as aboveground forest dynamics of growth and mortality. In contrast, aboveground forest biomass was more closely aligned with PC2 (Supplemental Fig. 1). We explore bivariate relationships between these different properties below.

### Soil properties

**Figure 2:**
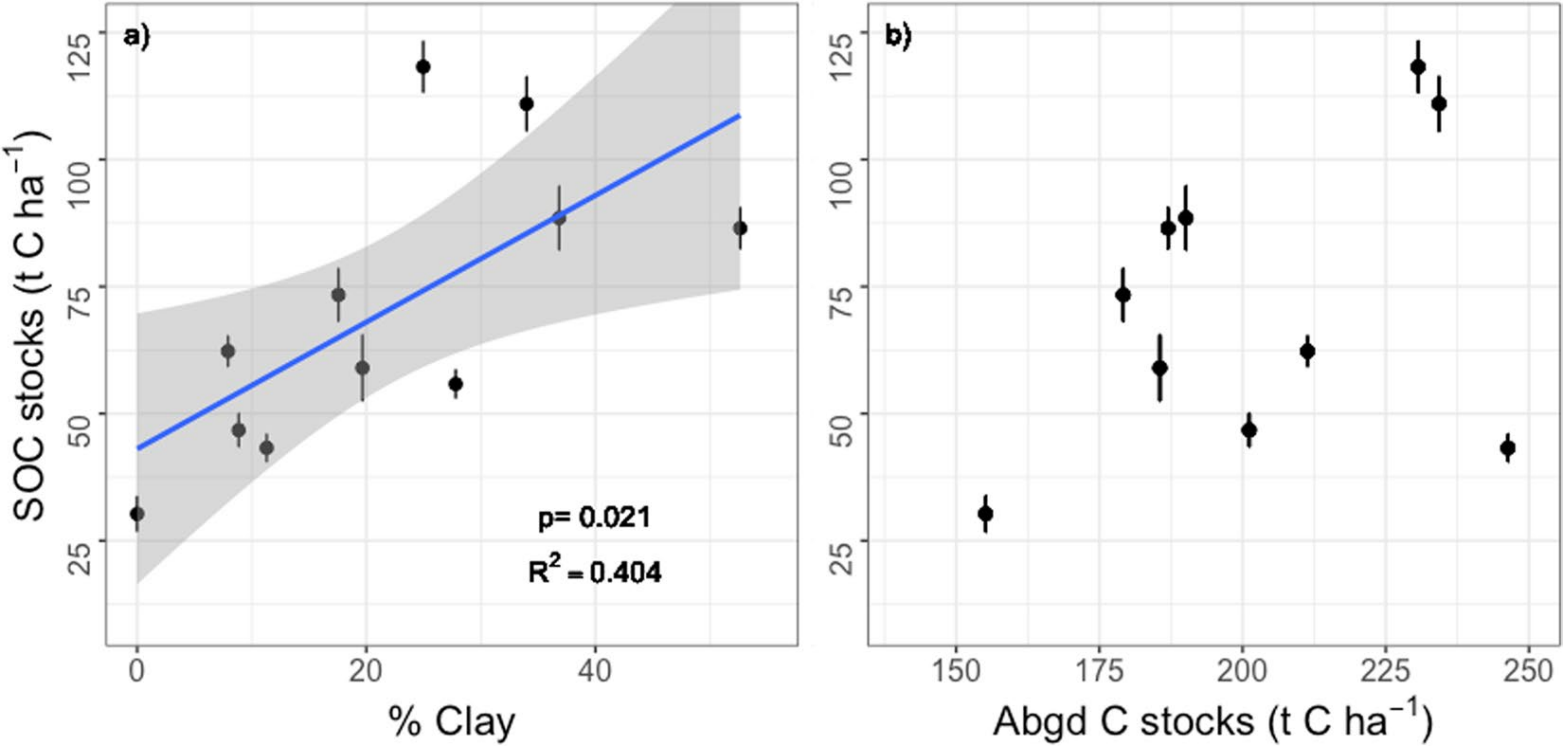
Mean soil organic carbon stocks (0–30 cm) relationship with (**a**) soil clay sized particle content, and (**b**) aboveground (Abgd) carbon stocks in woody biomass. Site TRB is missing from (**b**) because we lacked aboveground biomass measurements. Error bars are standard error.

Soil organic C stocks in the top 30 cm correlated positively with clay (<2 µm particle size) content (Fig. 2a; R^2^ = 0.404) across all 12 lowland tropical forest sites, and was also higher with a greater abundance of Fe and Al oxides in the soil (Supplemental Fig. 2). X-ray diffraction particle analysis showed that sites which were close to 100% sand in texture were comprised mainly of quartz, whereas sites with a mixture of sand, silt and clay sized particles were comprised of different proportions of quartz, Al and Fe oxides and hydroxides, and the 1:1 clay kaolinite (Table 1). The soils varied widely in texture, from sand at the LAU site to silty-clay at the MON, TRA, TIB and KAW sites (Table 1). Mineralogy also varied widely from 100% quartz at the LAU and SAL sites to over 80% clay and oxides (sum of gibbsite, iron oxides and kaolinite) at the KAW, MON, TIB and TRA sites (Table 1). This variation in soil physical characteristics likely stems from the diversity of geological substrates found across even a small geographic area in this Northern Amazon region, and their relief, that lead to differential weathering processes (Fig. 1).

**Figure 3:**
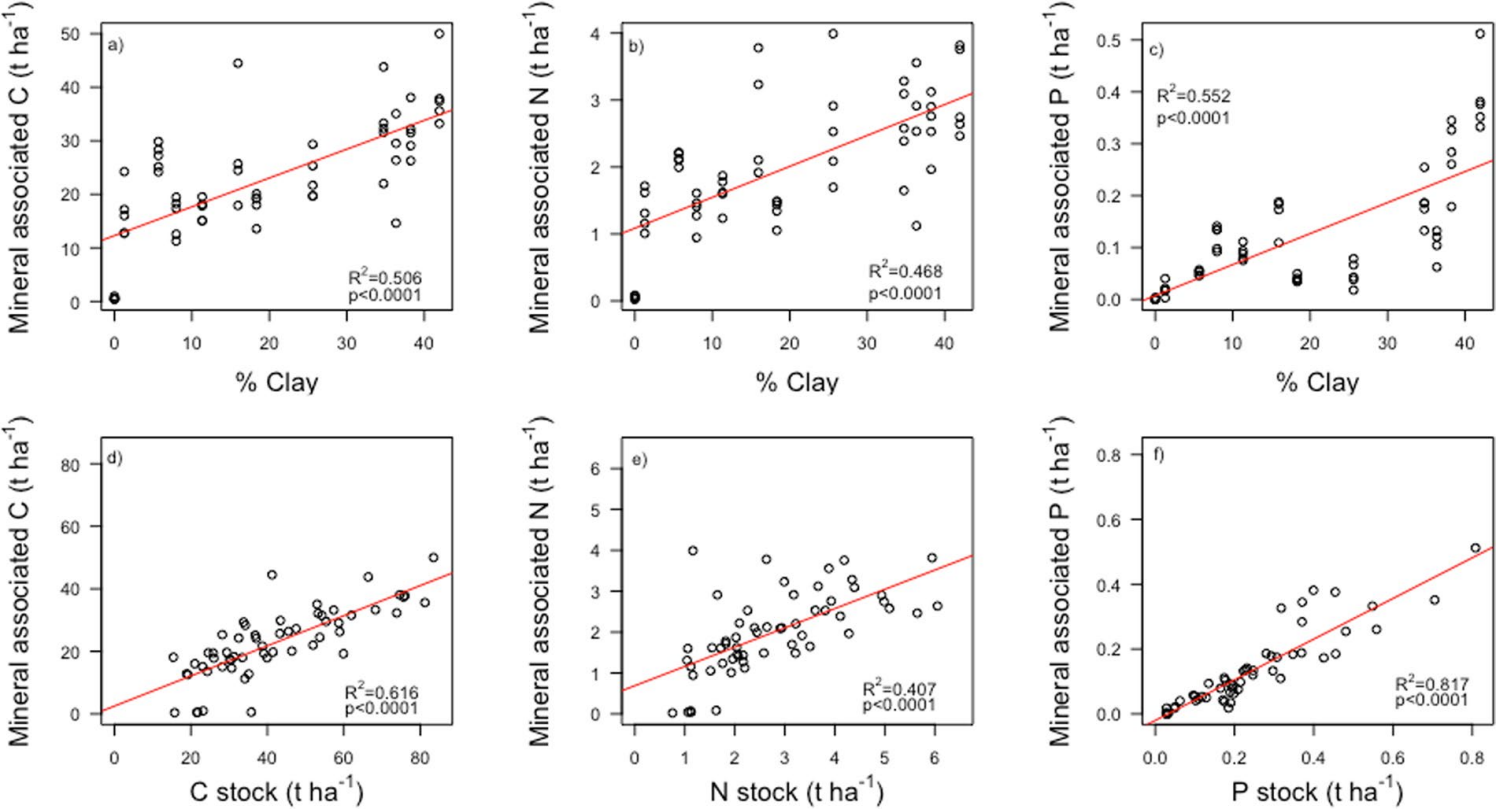
Mineral associated carbon (C) Nitrogen (N), phosphorus (P), and isolated by density and size fractionation and their association with, (**a**–**c**) clay (<2 µm) content of the soil, and (**d**–**f**) total elemental stocks in the top 15 cm of the soil. Stocks were calculated by multiplying elemental concentrations by bulk density.

Soil C stocks in the top 30 cm varied five-fold across our sites, from 30 to 118 tons C ha^−1^, and did not co-vary significantly with aboveground C stocks (Fig. 2). The coefficient of variation was 40% for soil C stocks across 11 sites and only 16% for aboveground C stocks across the 10 sites for which we had data, revealing a much larger variation between plots in soil than aboveground C stocks. In an analysis of soil organic matter fractions separated by density and size, we found positive Pearson correlations between mineral associated C, N, and P with clay-sized particle content (Fig. 3a–c) demonstrating the importance of mineral associations with fine particles in storing organic matter in these systems^32,33^.

**Figure 4:**
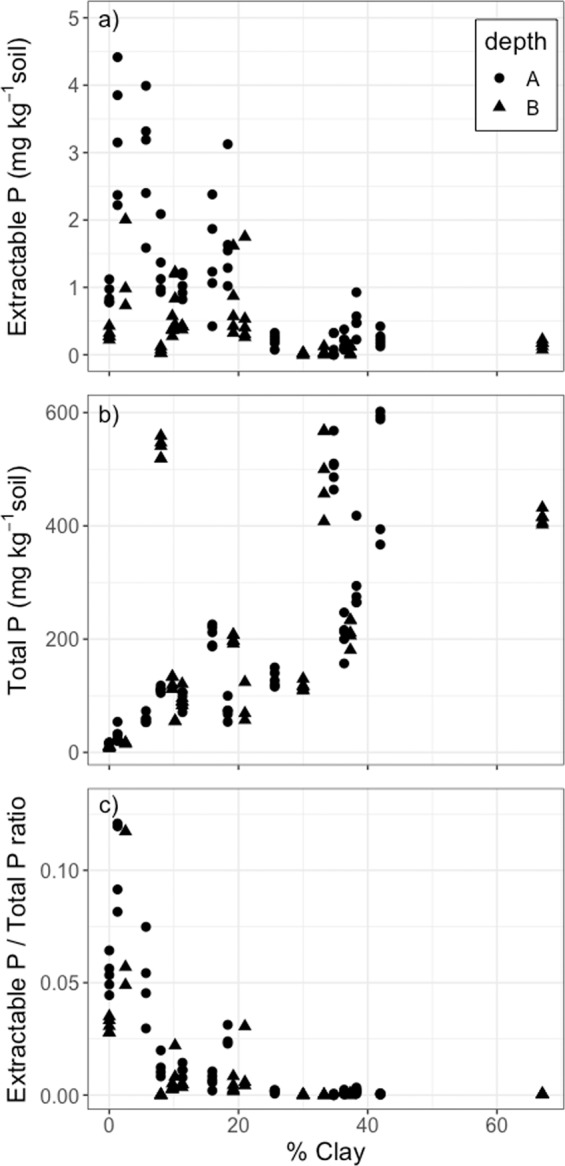
(**a**) Bray-extractable P, (**b**) total P, and (**c**) the ratio of Bray-extractable/total P versus soil % clay sized particle content for all soil samples. Symbols represent depths (A = 0–15 cm, B = 15–30 cm).

Soil extractable P (Bray-P)^20^ concentrations declined with increasing clay content (F_1,105_ = 6.76, p = 0.011; Fig. 4a) while, on the contrary, soil total P concentrations increased with clay content (F_1,100_ = 5.77, p = 0.018; Fig. 4b). Thus, the same Al and Fe oxides that adsorb organic matter and lead to higher soil C stocks and long-term storage also occlude phosphorus into unextractable forms^32^. Across our dataset, 87–100% of soil P was present in unextractable forms. We used the ratio of extractable-to-total P to measure the proportion of total P that was readily extractable (Fig. 4c). Although very little extractable P was measurable in any of our samples, the ratio of extractable-to-total P declined with soil clay content (Fig. 4c) up to a clay content of 25%, above which the ratio stabilized around zero. Below 25% clay fraction content, Al and Fe oxides and hydroxides were not or hardly present (Table 1). Above 25% clay content, where Al and Fe oxide contents were >16% (Table 1), the ratio of extractable-to-total P remained close to zero. Thus, 25% clay content appears to be a threshold, below-which increasing amounts of clay-sized particles is associated with decreasing proportions of P in extractable forms, and above-which increasing clay content does not affect the proportion of extractable P/total P. At the coarse end of this soil texture gradient, mineralogy and small amounts of clay and metal-oxide presence play a disproportionately large role in occluding extractable P into unextractable forms.

Using a density and size fractionation of the soil, we also found that P stocks correlated strongly with the amount of P in the heavy and <53 µm sized, mineral associated^34^, soil fraction (R^2^ = 0.82; Fig. 3f), further demonstrating the significant contribution of silt and clay sized particles total soil P content. The correlation between mineral associated P and soil P stocks was even stronger than the positive correlations between mineral associated C and N and soil C and N stocks (R^2^ = 0.616 and 0.407, respectively; Fig. 3d,e).

### Mycorrhizal fungi

**Figure 5:**
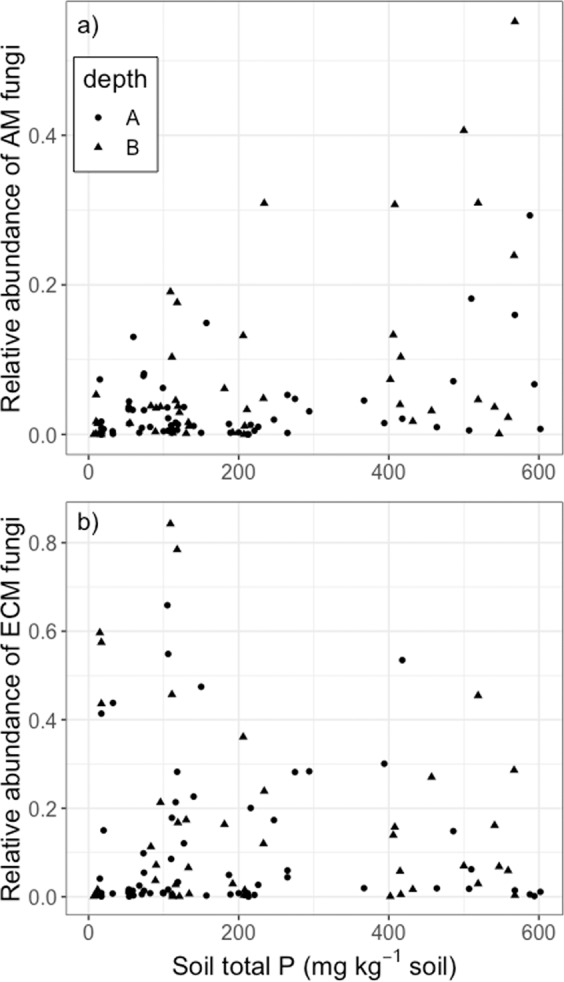
Relative abundance of (**a**) arbuscular mycorrhizal (AM), and (**b**) ectomycorrhizal (ECM) fungi, as measured with ITS DNA sequencing, and soil total P concentrations. Symbols represent depths (A = 0–15 cm, B = 15–30 cm).

The low concentrations of extractable P in all of the French Guiana sites (Fig. 4) implies that more complex P-uptake strategies are likely employed by the trees in these forests. One such strategy is mycorrhizal fungi associations. Internal Transcribed Spacer (ITS) DNA sequencing revealed the presence of both arbuscular mycorrhizal (AM) fungi and ectomycorrhizal (ECM) fungi across nearly all sites (Fig. 5). Contrary to our second hypothesis, the relative abundance of AM fungi increased with soil total P (p = 0.0084, Fig. 5a), while there was no significant relationship between ECM relative abundance and soil P (Fig. 5b).

### Forest dynamics

**Figure 6:**
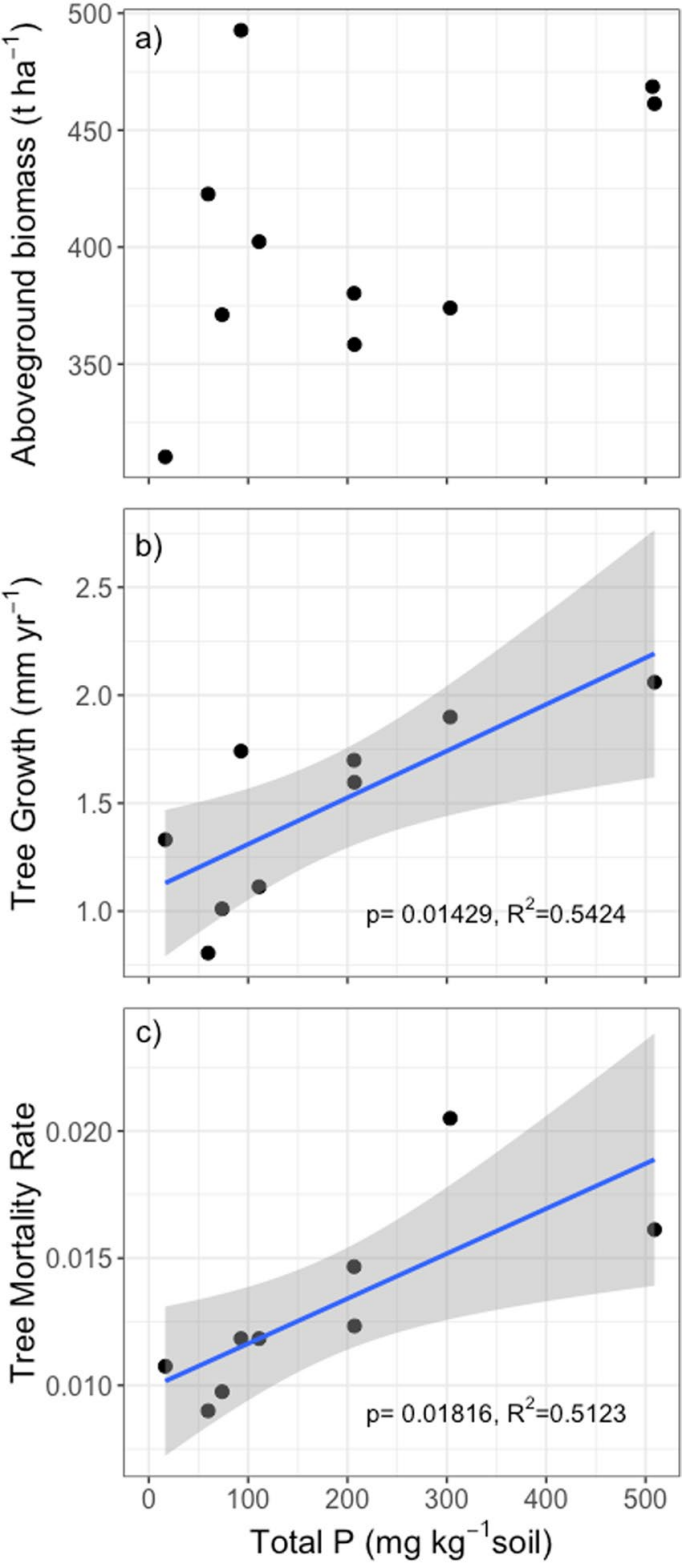
(**a**) Aboveground biomass (t ha^−1^), (**b**) tree growth rate (mm yr^−1^) for trees >10 cm diameter, and (**c**) tree mortality rates for trees >10 cm diameter from nine 1 ha forest plots across French Guiana plotted against soil total phosphorus concentrations in the top 0–15 cm of the soil. Blue lines are linear model fits and grey areas are 95% confidence intervals. In (**a**) there is no significant fit (p = 0.1676) so no fit is shown. One extra site is included in (**a**) because one year of aboveground biomass data was available, however multi-year dynamic growth and mortality rates were not available.

The dynamic forest properties of tree growth (R^2^ = 0.54) and mortality (R^2^ = 0.51) rates over time correlated positively with soil total P content, while the static forest property of aboveground biomass stock did not (Fig. 6). We found similar positive correlations between tree growth (R^2^ = 0.47), mortality (R^2^ = 0.63), and soil clay content, with no discernable correlation between aboveground biomass and clay content (Supplemental Fig. 3). Tree mortality can lead to canopy gaps and recruitment of new growth in Amazon forests, and thus can be used as an indication of forest turnover rates^35,36^. Leaf litter P concentrations also positively correlated with soil total P, while leaf litter C:N ratios declined with soil total P (Supplemental Fig. 4). Together, this indicates greater nutrient shedding via litter fall in clayey sites with high soil total P content^37^.

## Discussion

We found that both soil and aboveground forest properties were strongly related to soil mineralogy. According to the Microbial Efficiency Matrix Stabilization (MEMS) hypothesis, both the efficiency by which plant litter is decomposed by microbes (determined in large part by litter inputs, quality, and the microbial community), and the stabilization potential of the soil matrix, together determine soil C stocks^38,39^. In tropical soils, reactive clay surfaces, and Fe and Al (hydro)oxides coating other minerals, adsorb organic matter, leading to the formation of stable soil organo-mineral bonds and persistent soil organic matter^32,33^. The strong positive correlations we found between soil clay content and total soil carbon stocks (Fig. 2a) as well as mineral associated C, N and P stocks (Fig. 3) demonstrate the importance of clay- and silt-sized mineral surfaces, comprised mainly of gibbsite, Fe oxides (goethite, hematite) and kaolinite (Table 1), in storing C and nutrients in these lowland tropical forest soils. This is the first evidence for the MEMS hypothesis in the tropics, where rapid litter decomposition rates due to favorable climatic conditions make mineral matrix properties even more important to soil organic matter stabilization and nutrient availability^40^ than in colder regions where more undecomposed particulate organic matter can accumulate in soils^41^.

Mycorrhizal fungi were present in soil samples from all of these French Guiana sites (Fig. 5), reflecting the extent of this adaptive P uptake strategy in coping with the low P availability in this region. Arbuscular mycorrhizal fungi scavenge for inorganic nutrients released from saprotrophic microbial decomposition of organic matter, and can take advantage of the higher nutrient content and decomposability of litterfall in the clayey forests with high P retention^42,43^. The fact that AM fungi were relatively more abundant in sites with greater mineral-bound P, suggests that AM fungi may serve as an adaptive strategy to access the large proportion of P that is bound to soil minerals where the ratio of extractable-to-total P was almost zero^27,28^. Ectomycorrhizal fungi, which can access P from decomposing organic matter^28^ but are more adapted to providing plants with N, were present across all of the forest sites in our study, and did not vary with soil P content (Fig. 5). Similar patterns have been found across nutrient gradients in temperate forest sites in North America, where forest stands dominated by AM fungi were found to have more soil organic matter than ECM dominated stands^44^.

We found that the same belowground properties that led to greater soil C, N and P stocks—higher clay content and the presence of Al and Fe (hydro)oxides—also correlated with higher rates of aboveground tree growth and turnover (Fig. 6; Supplemental Fig. 3). This indicates that mineral surfaces that provide greater organic matter and P-retention capacity host faster cycling forests, although not forests with greater biomass. Reactive clay surfaces are more capable of retaining nutrients shed via litterfall and tree mortality in the system and could help to support nutrient retention in forests with faster turnover via mortality and growth. In clayey sites with high total soil P, we found that the faster growing trees also shed more nutrient rich litter. Due to the higher nutrient retention capacity of clayey soils, these sites could be more capable of benefiting from aeolian dust inputs of P blowing across the Atlantic Ocean from Africa^45–47^ than sandy sites lacking the mineral surfaces to retain added P resources in the soil. This finding could be used to predict which forest patches are expected to be responsive to nutrient additions based on their soil properties.

Slower growing trees with more conservative nutrient cycling strategies (i.e. slower growth, less mortality, nutrient-poor leaf litter) were found in sites with Quartz-rich, sandy, low total P soil, despite having a much greater proportion of soil P in an extractable form. Without reactive clay surfaces to retain P in the soil, these Quartz sand sites appear to have a more open nutrient cycle because extractable P could be easily leached and exported^48^. These sites were characterized by slow growing and longer-lived trees that avoid nutrient losses to the soil via litterfall. White quartz sand forests in the Amazon are known to favor the occurrence of endemic tree species^49^. This could reflect the slow growing species best adapted to these low nutrient soils with a low capacity for organic matter retention, cation exchange, water retention and structural support. Thus, our findings reveal that lower soil nutrient retention capacity was correlated with forests reflecting higher aboveground nutrient retention capacity (i.e. low litter nutrient loss, slow growth). These aboveground forest properties may reflect the adoption of nutrient conservative life history strategies, which promote low rates of P loss and can prevent the loss of P out of a leaky belowground soil matrix.

The fact that we found faster cycling forests (higher growth and mortality rates) correlated with soils with lower Bray extractable P^20^ calls into question the validity of Bray-P extracts as an indication of plant available P in tropical forests where plants have evolved adaptations to access non-extractable P^28^. Instead, extractions could be used as an indication of the leachability of nutrients from the soil matrix and an indication of the matrix potential to retain additional P from deposition or fertilization. This could provide a measure of the soil matrix potential^38^ to retain organic matter and nutrients in tropical forests where litter decomposes rapidly and heavy precipitation events increase the potential loss of resources from the plant-soil ecosystem. Our results indicate that soils with <25% clay content, which are mainly comprised of quartz (Table 1), may be more susceptible to nutrient leaching based on the evidence of P present in extractable forms, which was absent in soils with >25% clay (Fig. 4).

**Figure 7:**
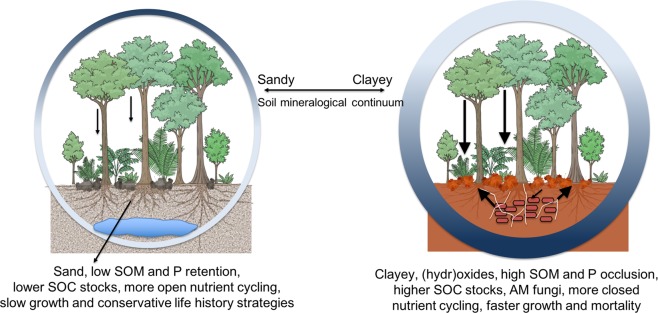
A simplified conceptual figure of the influence of soil properties on tree growth and mortality, but not biomass, across phosphorus-depleted tropical forests. Both forests have the same aboveground biomass, but different turnover rates and soil properties. At the sandy end of the soil continuum are forests with slower (narrower) nutrient cycling due to greater nutrient retention in the aboveground biomass (dark blue) based on slower growth, greater longevity, lower quality litter. At the other end of the spectrum are forests where the greater capacity of clay and (hydr)oxide-rich soils to retain phosphorus and organic matter support faster (wider) nutrient cycling forests. At clayey sites, nutrient recycling via decomposition (dark blue) is supported by a greater relative abundance of arbuscular mycorrhizal (AM) fungi.

In French Guiana, we found that lowland tropical forests with faster growth and mortality, on more P-rich soils, cycle C faster between the trees and the atmosphere due to faster growth rates (Fig. 6b) and higher leaf litter quality (Supplemental Fig. 4)^50^, while exhibiting similar aboveground stocks as low P, low growth forests (Fig. 6a). The distribution of clay content, which correlates positively with Al and Fe oxide and hydroxides, soil C stocks, and P stocks (Fig. 2a; Fig. 3; Supplemental Fig. 2), could be used as an initial indication of the distribution of faster cycling forests across the ancient lowland forests of the Guiana Shield (Fig. 7).

A growing body of research from Panama and across the Amazon points toward the importance of plant species diversity and individual species distributions as a means of disentangling tropical forest coupled belowground-aboveground patterns^9,30,51,52^. Species-specific analyses provide an important method for disentangling trait-based properties that bridge biodiversity with biogeochemistry. Here, we focus instead on stand-level patterns in French Guiana that could be used to overlay forest growth and mortality dynamics relevant to C cycling onto soil maps of the Guiana Shield region to upscale ecosystem exchange estimates to regional scales. Similar forest stand-level patterns of aboveground productivity reflecting soil properties were not found in Panama, possibly due to the younger, clayier, and less diverse range in soil texture an mineralogy of the Panamanian sites^9^. In a geologically diverse tropical region, such as the Guiana Shield, where large-scale species distributions are not well surveyed, the forest stand-level patterns of growth and mortality shown here provide a potentially scalable method of relating soil properties with aboveground forest dynamics.

Pedogenic processes occurring over millions of years across a variety of parent materials have led to a wide range in soil properties across the 200 km region studied here. We propose that the relatively undisturbed forests, which have developed over millennia^8^, have adopted life history strategies and symbioses with microbes to cope with these various soil properties that lead to more or less organic matter and nutrient retention in the ecosystem (Fig. 7). This could coincide with a sorting of tree species that are adapted to different positions along gradients from low to high nutrient scenarios^9^, and, along with drought tolerance, could explain differences in species composition between white sands forests and finer textured Ferralsols^53^. Moreover, across the twelve forest sites surveyed here, there was a gradient of soil properties ranging between the extremes in soil texture and P content with many intermediate scenarios. This provides great potential for different specialized niches to be filled, allowing the forests of the Guiana Shield to develop high diversity in both biota and life history strategies.

We found that although forest dynamics varied strongly with soil properties, aboveground biomass did not (Fig. 6), shedding light on the different biogeochemical niches^54^ within forests of similar aboveground biomass. The relationships between soil and aboveground forest traits revealed here support the conclusion that aboveground tropical forest dynamics may be driven in part by the capacity of soil to provide mineral-derived nutrients and retain organic matter across a broad range of soil substrates. If these patterns hold true across the region, better understanding of the geographic distribution of soil resources could be used to improve predictions of forest dynamics and the patterns of variability in forest dynamic responses to changing environmental conditions across the Guiana Shield region.

## Methods

### Site descriptions, soil and litter layer sampling and analyses

All of the forest sites were located in the Northern region of French Guiana and span various geological substrates (Fig. 1). Soils are mainly classified as Oxisols, Ultisols and Spodosols. Mean annual precipitation ranges from approximately 2,400–4,000 mm yr^−1^ (Table 1). We created the map in Fig. 1 using ArcGIS, Version 10.3^55^ and shapefiles of the French Guiana boundary, the GPS coordinates of our sampling sites, and maps of geological substrate.

We selected 12 lowland tropical forests with no known history of logging with long-term forest monitoring in French Guiana. Ten out of the 12 sites were in the center of 1 ha forest monitoring plots of the Guyafor network. In July, 2015, we sampled 5 replicate spots within a 20 m × 20 plot. The soil plots were established in the middle of each forest site for the 10 forest monitoring sites. At each spot we first collected the litter layer within a 20 cm^2^ area. From these same five spots, we then took three soil cores, 3 cm in diameter, down to 30 cm and divided the core into a 0–15 cm topsoil and a 15–30 cm sub-soil sample. Since soil horizonation was different at different sites, we chose these two standard depths to remain consistent across sites. We composited the three cores from each spot to end up with 5 replicate soil samples from each depth for chemical, textural, biological, and mineral analysis. We took an additional 8 cm diameter core at each of the 5 replicate spots from the 0–15 and 15–30 cm depths, taking care to collect the exact volume of the core, for measurement of soil bulk density.

A subsample of the soil sampled for chemical analysis was frozen as soon as possible (within six hours) and then freeze-dried prior to 2 mm sieving. The freeze-dried samples were used for DNA extraction (0.25 g, Powersoil DNA isolation kit, Mobio, Carlsbad CA, USA). Two negative controls were produced by performing all the same steps from DNA isolation onwards to account for potential lab-contamination. One µl of the resulting solution was used as template in a 25 µl PCR containing 0.25 U of Phusion High-Fidelity polymerase (Thermo Scientific, Waltham, MA, USA) in a solution containing 1x PCR buffer, 200 µM dNTP’s, and 200 nM general fungal primers ITS1f and ITS2 augmented with multiplexing barcodes as in^56^. We used a primer set targeting total fungi because it allows us to (1) simultaneously detect both groups of mycorrhizal fungi (i.e. AM and ECM), and (2) to derive a semi-quantitative estimate of relative abundance of both functional groups. PCR conditions were as follows: initial denaturing at 98 °C for 30 s, followed by an additional round at 98 °C for 30 s, annealing at 55 °C for 30 s, extension at 72 °C for 30 s, the latter three steps of which were repeated for a total of 30 times with an additional final extension step of 72 °C for 10 min. Successful amplification products were pooled and cleaned (Beckman Coulter, Brea, CA, USA), quantified using a Qubit fluorometer (Thermo Fisher Scientific, Waltham, MA, USA), and pooled to equimolar concentrations. This pool was additionally loaded onto a 1% agarose gel and excised to remove remaining primers, cleaned using the QIAquick Gel Extraction Kit (Qiagen, Venlo, the Netherlands) and again quantified. This product was sequenced on an Illumina MiSeq using V2 chemistry (2 × 150) for 300 cycles in the forward direction and 12-cycles for indexing, in presence of a 20% spike of PhiX to ensure sufficient diversity.

Sequences were analysed using the UPARSE pipeline^57^, with the following steps/settings: sequences were trimmed to 250 bp and quality filtered according to a maximum estimated error of 0.5% leaving a total of 4,424,967 non-singleton sequences, which were clustered to operational taxonomic units (OTU’s) at 97% OTUs similarity. Chimera’s were filtered *de novo* as well as through using the UNITE^58^ resulting in a total of 22,572 non-chimeric OTUs. OTUs were aligned to all fungal representative species in the UNITE and INSD database using the BLAST algorithm selecting hits with the lowest E-value using a threshold of max E value of 1 * 10^−36^ for inclusion (e.g. Waring, *et al*.^59^). Because this criterium is very stringent for lineages with high ITS diversity such as Glomeromycota, we additionally analysed sequences at an E value cutoff of 1 * 10^−20^, but with a minimal alignment length of 150 bp to assign further Glomeromycota. We removed one fungal OTU that occurred in one of the negative controls. Number of reads per sample were then rarefied (*rarefy*) in VEGAN^60^ to 2,629 reads which retained a total of 5,132 fungal OTUs, and proportions of AM or ECM fungal reads calculated. The raw sequences were deposited in the National Center for Biotechnology's Information's (NCBI's) Sequence Read Archive database under the accession no. PRJNA603474.

All litter samples were oven dried at 45 °C for 72 hours and weighed to determine litter dry mass. The dried samples were then ground and sub-sampled in duplicate for analysis of %C and %N by dry combustion (Macro Elemental Analyzer, model vario MAX CN, Hanau, Germany). The average of the two %C and %N values is reported, and deviation between samples was <5%. Litter was also measured for total P content on a continuous flow analyzer (SAN++, SKALAR, Breda, NL) after digestion with sulphuric acid, selenium and salicylic acid^61^.

The soil samples for chemical analysis were sieved to 2 mm and oven dried at 65 °C for 48 hours in order to stabilize them for storage (<1 month) prior to analysis. Half of the sample was ground and used for analysis of %C, %N. The other half of the 2 mm sieved soil was used to measure pH (in KCl, using a Calibration Check pH/mV/ISE/Temerature Benchtop Meter, Hanna Instruments, Temse, Belgium), extractable P using the Bray P method^20^ and total P content via acid extraction^62^. All phosphorus extracts were analyzed on a continuous flow analyzer (SAN++, SKALAR, Breda, NL).

The large bulk density cores were sieved to 4 mm and oven dried at 100 °C for 48 hours. We determined the volume of the rocks and roots >4 mm and weighed the oven dried soil <4 mm to calculate soil bulk density. Soil C and N stocks were determined by multiplying bulk density by C and N concentration on a per sample basis. At one site, Sal, the soils were so sandy and unconsolidated that we were unable to collect accurate 15–30 cm cores for bulk density, and thus were unable to calculate soil bulk density and stocks.

Soil texture was determined via the hydrometer method on oven dried soil samples sieved to <2 mm^63^. A composite soil sample from the five 0–15 and 15–30 cm soil samples from each forest site was used for soil texture. Briefly, we pre-treated all soil samples with 30% H_2_O_2_ solution to remove all organic matter. After oven drying, we then shook 40 g of soils with 5% Sodium Hexametaphosphate solution to disperse all aggregates. We determined the percentage of sand sized particles (53–2000 µm) via sieving and determined silt sized (2–53 µm) and clay sized (<2 µm) particles via solution density after 6 hours of settling at 20 °C. In our hydrometer-based soil texture analysis, clay content is defined by particles <2 µm in size and thus contains both clay and metal-oxide minerals.

An aliquot of the same composite soil sample used for soil texture analysis was also used for semiquantitative X-ray diffraction (XRD) particle analysis of soil mineral composition. 2 mm sieved soil samples were ground into powder. Diffractograms of the sample powder were obtained with a PANalytical X’Pert PRO MPD-DY 3197 diffractometer of the Serveis Científico-Tècnics (SCT) of the Universitat de Barcelona. Results were analyzed using the X’Pert HighScore Plus software.

We employed a physical soil organic matter fractionation scheme in order to separate the particulate soil organic matter from the primary organo-mineral complexes^34^. After dispersion with glass beads, soil from the 0–15 cm depth of five oven dried, 2 mm sieved, soil samples from all forest sites was fractionated into three pools according to the method described in Soong and Cotrufo^64^. Briefly, a 1.85 g cm^−3^ sodium polytungstate solution was used to separate the particulate organic matter (<1.85 g cm^−3^) from the heavy fraction. The heavy fraction (>1.85 g cm^−3^) was then sieved to separate the sand sized fraction (>53 µm) from the silt & clay sized fraction (<53 µm). Total mass recovery of the three oven dried fractions was +/− 5% from the starting soil mass. The particulate organic matter, sand and silt & clay fractions were ground and analyzed for %C and %N in duplicate by dry combustion elemental analysis (Flash 2000 series CN analyzer, Thermo Scientific, Darmstadt, Germany). Any remaining sample was then analyzed for total P^61^ on a continuous flow analyzer (SAN++, SKALAR, Breda, Neatherlands).

The data from our July 2015 soil and leaf litter sampling campaign was compared with forest structure and dynamics data from ten long-term forest monitoring sites described in Grau *et al*.^6^ and Desprez, *et al*.^65^. Our 20 × 20 soil sampling plots were located in the center of these 1 ha forest monitoring plots that were censused for tree species composition and monitored every 2–5 years. Briefly, biomass of each tree with diameter at breast height (DBH) >10 cm was estimated using a pantropical allometric equation^66^ and summed for all living trees to obtain plot level total aboveground biomass. Aboveground C stocks were calculated by multiplying aboveground biomass by 0.5, the approximate C concentration of woody biomass^67,68^. Growth rate was calculated as the mean increase in DBH (mm yr^−1^) and mortality rate was estimated using estimators of instantaneous mortality^6,69^ i.e. accounting for the initial number of trees in a plot, the number of survivors in a plot, the number of dead trees per plot and time between two consecutive censuses, as described by Grau *et al*.^6^. Growth and mortality data were available was not available for the site named KAW, where only aboveground biomass data was available.

### Data analysis

We conducted a principal component analysis (PCA) using the ‘Vegan’ package in R 3.2 to identify the relationship between variables of soil, fungi, and forest structure among sites. Based on initial data exploration, we chose to include the soil variables of clay content, Bray P (extractable P), soil C stock, Fe and Al oxide relative content, relative abundance of arbuscular mycorrhizal fungi, relative abundance of ectomycorrhizal fungi, and total P content, along with the forest variables of growth rate, mortality rate, and aboveground C stock. We used data from the nine sites that contained the complete suite of data needed for this analysis.

We used linear regressions for bivariate analysis of soil properties such as soil organic carbon (SOC) stock, % Clay, Total P, Bray-P, surface litter C:N, surface litter P and relative abundance of AM fungi. We analyzed the effect of %Clay content on SOC stocks, total P and Bray-P, the effect of soil total P on litter C:N, litter C:P, litter P, green leaf P, and relative abundance of AM fungi and the effect of total P on relative abundance of AM fungi using restricted maximum likelihood analysis with % Clay as a fixed effect and depth and replicate nested within Site as random effects using the R package ‘nlme’^70^. We used logarithmic or square root transformations to fit the assumptions of these models when needed (i.e., for soil total P *versus* % Clay, Bray-P *versus* % Clay, and AM fungi *versus* soil total P). We utilized Michaelis-Menten equations to explore the non-linear relationships between soil total P, extractable P and the ratio of extractable-to-total P against soil clay content. These models were written in Stan^71^ with inference using Hamiltonian Monte-Carlo algorithms and implemented in Rstudio using the Rstan package^72^.

For the nine sites with multiple years of forest data, we tested the effect of soil total P in the top 0–15 cm depth from the 2015 soil sampling measurements on forest growth and mortality rates measured from 2000–2016 using restricted maximum likelihood with soil total P as a fixed effect and site as a random effect. One additional site (Kaw) had only one year of aboveground biomass data but no information on productivity and mortality. We used the mean value of the five soil samples from the 0–15 cm depth at each site to compare to the singular values of aboveground biomass, tree growth and tree mortality for each site^6^. We also tested the effect of soil total P in the top 0–15 cm depth from the 2015 soil sampling on aboveground biomass for all ten sites using the same model.

## Supplementary information

Supplementary Figures.

## Data Availability

Once accepted for publication, the data will be made available on an open repository supported by the University of Antwerp.
