# Supplementary material for: Soil properties explain tree growth and mortality, but not biomass, across phosphorus-depleted tropical forests
Source: Sci Rep. 2020 Feb 10;10:2302. doi: 10.1038/s41598-020-58913-8 (PMC7010742; doi:10.1038/s41598-020-58913-8)

**Soil properties explain tree growth and mortality, but not biomass, across phosphorus-depleted tropical forests**

Jennifer L. Soong^1,2^, Ivan A. Janssens^2^, Oriol Grau^3,4^, Olga Margalef^3,4^, Clément Stahl^5^, Leandro Van Langenhove^2^, Ifigenia Urbina^3,4^, Jerome Chave^6^, Aurelie Dourdain^7^, Bruno Ferry^8^, Vincent Freycon^9,10^, Bruno Herault^10,11^, Jordi Sardans^3,4^, Josep Peñuelas^3,4^, Erik Verbruggen^2^

^1^ Climate and Ecosystem Science Division, Lawrence Berkeley National Laboratory, Berkeley, California, USA

^2^ Department of Biology, University of Antwerp, Wilrijk, 2610 Belgium

^3^ Center for Ecological Research and Forestry Application, 08193 Cerdanyoladel Vallès, Catalonia, Spain

^4^ Consejo Superior de Investigaciones Científicas, Global Ecology Unit CREAF-CSIC-UAB, 08193 Cerdanyola del Vallès, Catalonia, Spain

^5^ INRA, UMR Ecology of Guiana Forests (Ecofog), AgroParisTech, Cirad, CNRS, Université de Guyane, 19Université des Antilles, 97387 Kourou, France

^6^ Paul Sabatier University, CNRS, Toulouse, France

^7^ CIRAD, UMR Ecology of Guiana Forests (Ecofog), AgroParisTech, INRA, CNRS, Université de Guyane, Université des Antilles, 97387 Kourou, France

^8^ Université de Lorraine, AgroParisTech, INRA, Silva, 54000 Nancy, France

^9^ CIRAD, UPR Forêts et Sociétés, F-34398 Montpellier, France

^10^ Forêts et Sociétés, Université de Montpellier, CIRAD, Montpellier, France

^11^ Institut National Polytechnique Félix Houphouët-Boigny, Yamoussoukro, Ivory Coast

**Supplemental Information**

**Supplemental Figure 1.** Principle component analysis of soil properties, fungi communities, and aboveground forest properties that from nine French Guiana forest sites. Individual forest sites are indicated by different colors and ellipses.

**
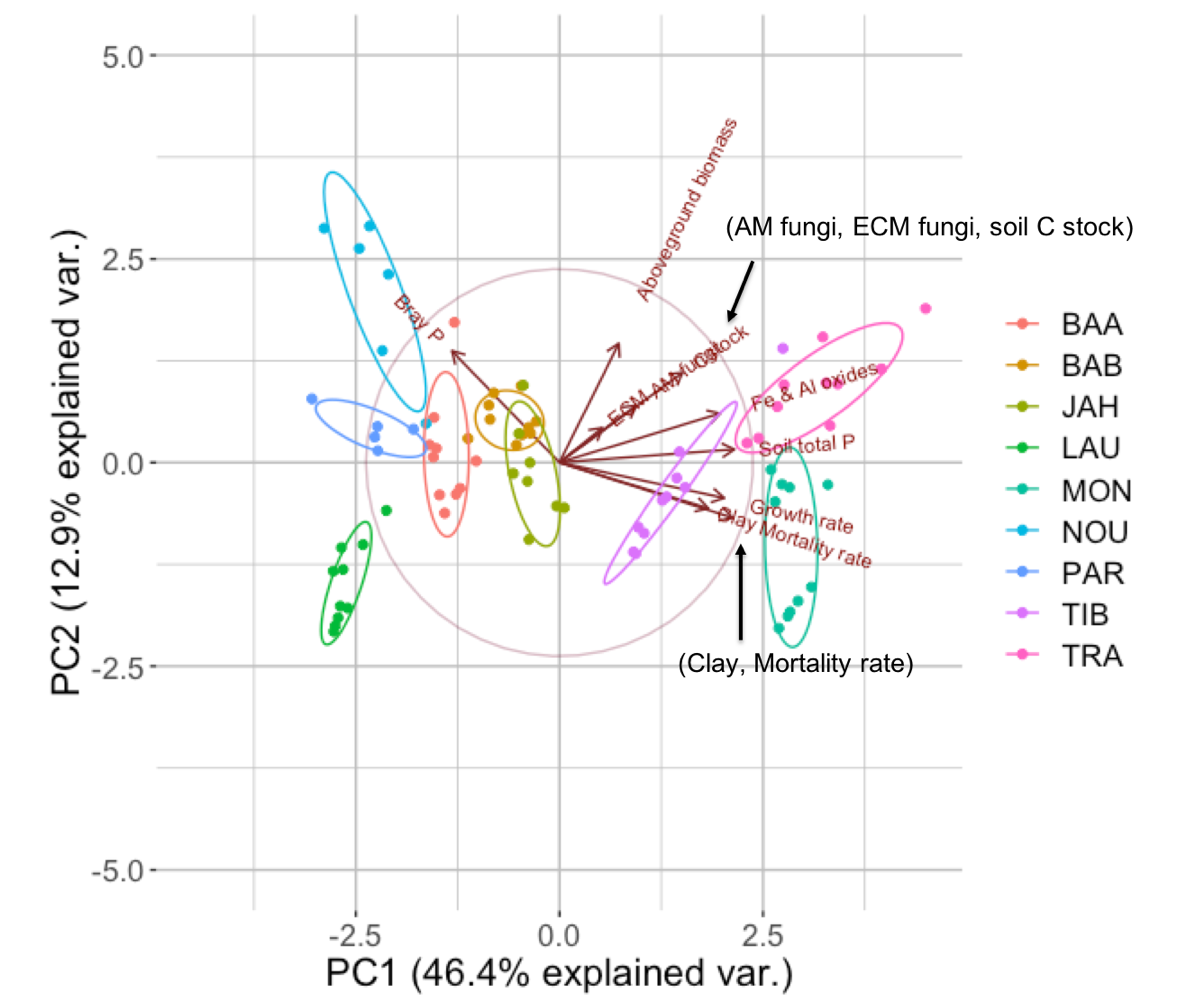
**

**Supplemental Figure 2.** Ternary diagram of the mineralogical composition of quartz, metal-oxides and hydroxides (Gibbsite, Goethite, Hematite) and kaolinite from soil samples as determined using XRD analysis. Points are individual samples with circles showing the A depth (0-15 cm) and triangles representing the B depth (15-30 cm). The color scale indicates mean percent carbon values of five samples, as indicated by the color bar. Carbon content tends to increase especially with the presence of Fe and Al oxides and hydroxides.

**
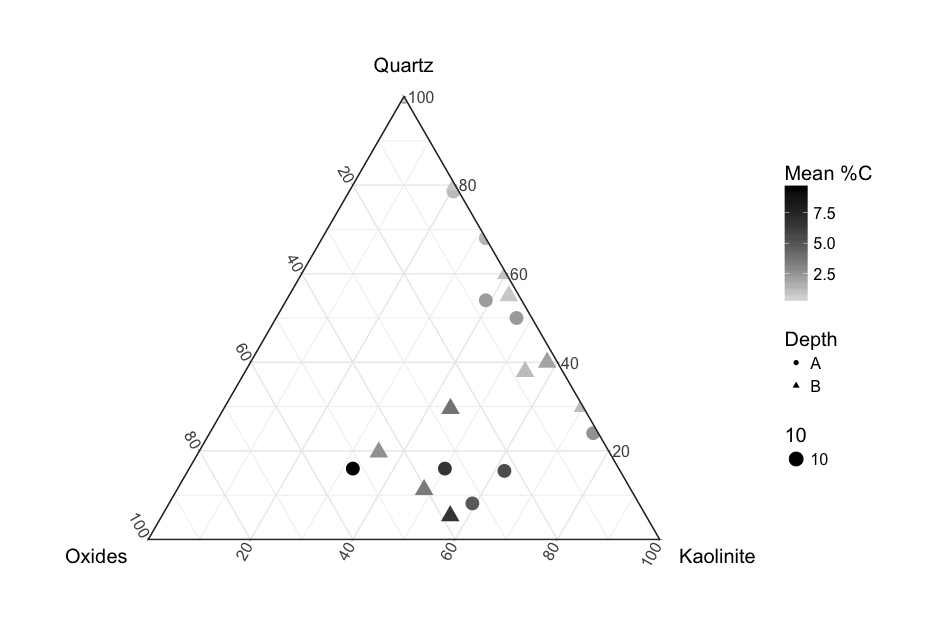
**

**Supplemental Figure 3.** a) aboveground biomass (tons ha^-1^), b) tree growth rate (mm yr^-1^) for trees > 10 cm diameter, and c) tree mortality rates for trees > 10 cm diameter from nine 1 ha forest plots across French Guiana plotted against soil clay content in the top 0-15 cm of the soil. Blue lines are linear model fits and grey areas are 95% confidence intervals. In a) there is no significant fit so no fit is shown. One extra site is included in a) because one year of aboveground biomass data was available, however multi-year dynamic growth and mortality rates were not available.


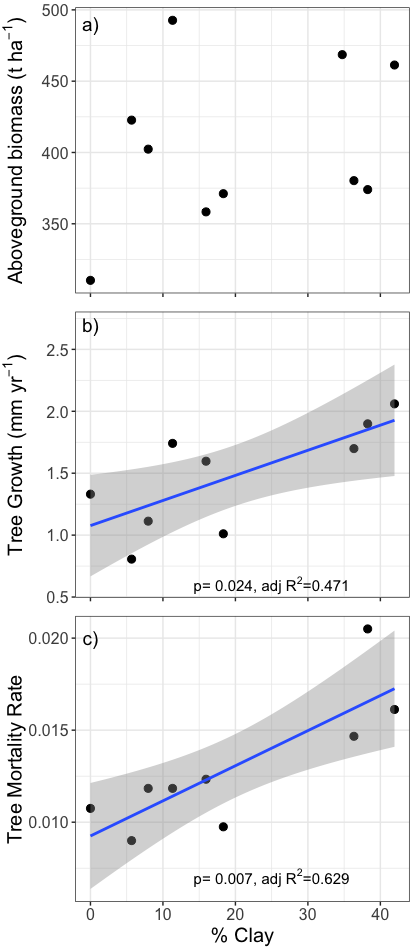


**Supplemental Figure 4.** Leaf litter nutrient quality as determined by, a) C:N ratio, and b) % P content regressed against soil total P concentrations**.** The blue line is a linear model fit and grey areas are 95% confidence intervals


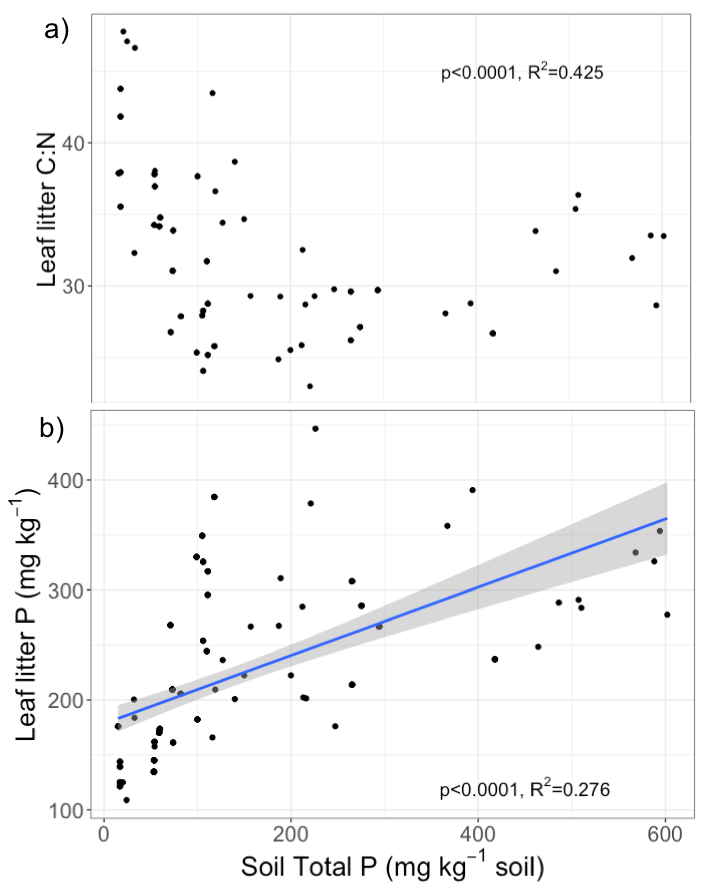

Supplement: Supplementary file 1 — Supplementary Figures. [file 41598_2020_58913_MOESM1_ESM.docx]
